# 953. Implementation Outcomes in an Antibiotic Stewardship Program (Kicking CAUTI) in Four Veterans Hospitals Correlated with Clinical Outcomes

**DOI:** 10.1093/ofid/ofac492.796

**Published:** 2022-12-15

**Authors:** Eva Amenta, Larissa Grigoryan, David J Ramsey, Jennifer Kramer, Annette Walder, Andrew Chou, John Van, Anne Sales, Aanand Naik, Barbara Trautner

**Affiliations:** Baylor College of Medicine, Houston, Texas; Baylor College of Medicine, Houston, Texas; Michael E. DeBakey Veterans Affairs Medical Center, Houston, Texas; Baylor College of Medicine, Houston, Texas; Baylor College of Medicine, Houston, Texas; Michael E. DeBakey Veterans Affairs Medical Center, Houston, Texas; Baylor College of Medicine, Houston, Texas; University of Missouri, Colombia, Missouri; The University of Texas Health Science Center at Houston, Houston, Texas; Michael E. DeBakey Veterans Affairs Medical Center / Baylor College of Medicine, Houston, TX

## Abstract

**Background:**

One of the major barriers to scale up of antibiotic stewardship interventions is the difficulty of engaging already overtaxed personnel. As part of a multisite antibiotic stewardship project to decrease antibiotics treatment of asymptomatic bacteriuria, we explored how to measure local implementation efforts, and what dose of the intervention was necessary to improve clinical outcomes.

**Methods:**

The intervention was implemented in 4 different sites from February 2019 through May 2020. We chose 3 measures of implementation: the number of intervention delivery sessions (adoption), total number of health care professionals reached (penetration), and minutes spent in delivery of the intervention (adoption). Local site champions kept activity logs. Antibiotic prescriptions were included if ordered within one calendar day prior or two days after a urine culture was ordered on the same patient. Correlation between implementation measures and clinical outcomes (number of urine cultures ordered, days of antibiotic treatment (DOT), and length of antibiotic treatment (LOT)) was calculated using the mixed linear models method.

**Results:**

Overall, the minutes spent in delivery ranged from 2567 minutes at the most engaged site to 679 in the least engaged site (Figure 1). The number of healthcare professionals reached ranged from 798 to 433, and the number of sessions delivered ranged from 240 to 45. Minutes spent in delivery was inversely correlated with two of our three clinical metrics, DOT (R -0.3, P=0.04) and LOT (R -0.3, P=0.02); minutes spent and urine cultures were not significantly correlated (Table 1). We did not find a significant relationship between the number of intervention delivery sessions or total number of health care professionals reached and any of the clinical outcomes.

**Figure 1:**
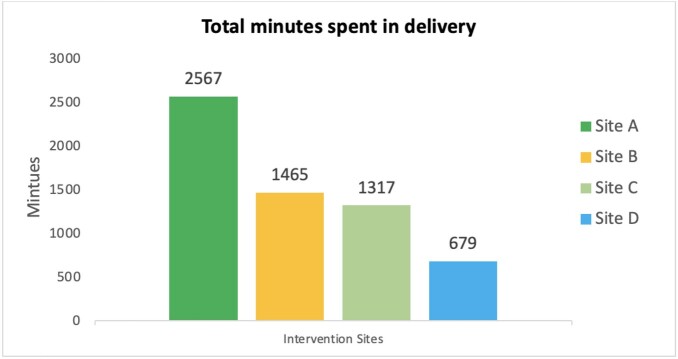
Total Minutes Spent in Delivery of the Intervention Across Four Intervention Sites

**Table 1:**
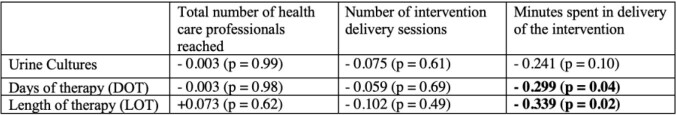
Correlation Coefficients (with p-values) Comparing Implementation Outcomes with Clinical Outcomes (bolded results are statistically significant)

**Conclusion:**

We found a significant inverse correlation between the number of minutes a local site champion spent delivering the antibiotic stewardship intervention and antibiotic use, both DOT and LOT. In other words, more time spent delivering the intervention locally was associated with a decrease in antibiotics ordered. Our implementation metric (adoption) is scalable and readily adaptable to large antibiotic stewardship dissemination projects.

**Disclosures:**

**Larissa Grigoryan, MD, PhD**, Rebiotix Inc: Grant/Research Support **Barbara Trautner, MD, PhD**, Genetech: Advisor/Consultant.

